# Qualitative study of presumptive treatment of childhood malaria in third tier tertiary hospitals in southeast Nigeria: a focus group and in-depth study

**DOI:** 10.1186/1475-2875-12-436

**Published:** 2013-12-01

**Authors:** Maduka D Ughasoro, Chinedu C Okoli, Benjamin SC Uzochukwu

**Affiliations:** 1 Department of Paediatrics, University of Nigeria Enugu Campus, Enugu, Nigeria; 2 Health Policy Research Group, University of Nigeria Teaching Hospital, Enugu, Nigeria; 3 Department of Surgery, Nnamdi Azikiwe University Teaching Hospital, Nnewi, Nigeria; 4 Department of Community Medicine, University of Nigeria Enugu Campus, Enugu, Nigeria

**Keywords:** Presumptive treatment, Childhood malaria, Tertiary hospitals, Nigeria

## Abstract

**Background:**

Presumptive treatment of childhood-malaria (PTCM) is common in Nigeria. Delayed laboratory result is blamed, with little attention on patients’ and providers’ roles. This study aimed to determine patient, provider and laboratory attributes that sustain PTCM in Nigeria.

**Methods:**

Data collection was from focus-group discussions for parents/guardians, and in-depth interviews involving providers and laboratory scientists in two tertiary hospitals.

**Results:**

All parents/guardians agreed to a malaria test. Majority accepted to come back later for full treatment, provided that some treatment was commenced. Majority affirmed that their interests are on their children’s improvement.

The providers practice presumptive treatment of childhood malaria, for the following reasons: (1) malaria is endemic and should be suspected and treated; (2) microscopy takes two days to be available and parents want immediate treatment for their children, thus delay may lead to self-medication; (3) relying on results for decision to treat creates an impression of incompetence; (4) rapid diagnostic test kits (RDTs) are not available in the consulting rooms and there is doubt about their reliability; (5) patients have already wasted time before being reviewed, so wasting more time on investigation is not advisable; (6) withhold of malaria treatment may be feasible in suspected uncomplicated malaria, but if severe, then anti-malarial treatment has to start immediately.

Interviews of laboratory scientists showed that (1) malaria microscopy test cannot be urgent; it is done in batches and takes 24 hours to be ready; (2) a request of malaria test with other investigations on the same form, contributes to the delay; (3) RDTs are unavailable in the facilities.

**Conclusions:**

Provision of RDTs is the only feasible means to treatment of confirmed malaria at the time healthcare providers review a patient on day zero. In facilities that depend on microscopy; a common practice in resource poor countries, healthcare providers can depend on parental willingness to return later for full medication, to commence adjunctive care with antipyretics and multivitamins for uncomplicated malaria. In complicated malaria, supportive care - intravenous fluids, blood transfusion, oxygen therapy - can be commenced while awaiting the inclusion of anti-malarial drugs when the diagnosis of malaria is confirmed.

## Background

In April 2000, the Roll Back Malaria (RBM) programme was launched in Abuja, Nigeria with the sole aim of reducing the burden of malaria in sub-Saharan Africa [1]. One of its strategies is early diagnosis and treatment of malaria [2]. Due to high prevalence of malaria in these areas, and its associated high mortality in children, it was advocated that an anti-malarial can be given based on the presence of fever [3]. Another reason for this is the anticipated high mortality that will occur especially in children if anti-malarial therapy is delayed for malaria test results to be available, the problem of lack of laboratory support is expected more in the primary health care facilities. Furthermore, it was argued that since the prevalence of malaria was high in most sub-Saharan regions, and first tier facilities are amongst the first source of care for the patients, presumptive treatment will be cost-effective if practiced there [4]. Studies have shown that if 70% or more of fever cases are malaria fever, presumptive treatment is more cost-effective than prior microscopy or rapid diagnostic test (RDT) when artemisinin-based combination therapy is the anti-malarial in usage [5,6].

It is 13 years since the introduction of RBM programmes in sub-Saharan Africa. Studies have shown a decline in the burden of malaria in these endemic areas [7,8], with a concomitant reduction in the proportions of fever that can be attributed to malaria. This was supported by a report by Tagbo *et al. *[9] where they documented positive malaria parasitaemia of 29.2% amongst children with history of fever in a tertiary clinic. Also, advancements have been made in the area of malaria diagnosis with the introduction of simple and easy to apply malaria RDT kits [10,11]. Presumptive diagnosis and treatment of malaria is still being practiced not only in the primary health care facilities, but also in the tertiary hospitals [12-14]. Considering the prevailing statistics, continued practice of presumptive treatment will not only lead to over-diagnosis of malaria, but also a lot of missed diagnosis of other illnesses that have both immediate and long-term morbidity and mortality. Other benefits of laboratory-confirmed diagnosis and treatment of malaria in children are: reduction of drug wastage, avoidance of adverse drug events, and evasion of imminent loss of confidence in anti-malarial drugs by the public due to use of anti-malarial in cases that are not malaria cases.

The current guideline, has changed its stand on presumptive treatment of all childhood fever in sub-Saharan regions with anti-malarial drugs [15], the global reduction of malaria and the introduction and deployment of malaria rapid diagnostic test kits (RDTs) to health facilities has made the practice of presumptive treatment less fashionable particularly in tertiary hospitals, where most patients are likely to have received some form of self-medication before presenting, which almost always includes an anti-malarial [16]. The doubt about the adequacy of the dosage of medication administered is erased by the current innovation of clearly stating the dosage in terms of weight and age on the packet of the drugs by pharmaceutical firms. In view of this, it is most likely that adequate anti-malarial therapy had already been taken before presenting to the health facility. Therefore, presumptive practice in tertiary clinics is not permissible, but various reports have continued to document a high practice of presumptive treatment of malaria in tertiary hospitals [12].

It is still surprising that even with the introduction of RDTs, which are a good alternative to microscopy [17,18], the expected improvement in the practice of confirmation of malaria is not being reported. This gives an insight that there may be more factors to presumptive treatment of malaria than difficulties with microscopy test for malaria. This study is aimed at determining the perspectives of patients, health-care providers and laboratory scientists regarding the confirmation of malaria in a tertiary hospital. The results might be useful in designing appropriate interventions that will improve the practice of confirmed malaria treatment in tertiary hospitals in Nigeria.

## Methods

### Study area

The study was conducted in two tertiary hospitals: University of Nigeria Teaching Hospital, Enugu, and The Federal Medical Center, Umuahia, both in southeast Nigeria. Both hospitals are in the guinea-savanna forest region of Nigeria where malaria transmission is stable all year round. The two hospitals offer specialist medical care and also have Residency training programmes in paediatrics.

### Study design

The study was a qualitative, descriptive cross-sectional study. The University of Nigeria Teaching Hospital, Enugu and the Federal Medical Centre Umuahia, were selected by simple random sampling from a sample frame of four tertiary hospitals in Enugu and Abia States.

In-depth interview (IDI): A list of all the residents and consultants in each of the selected hospitals were compiled, as well as the laboratory scientists/technicians that currently work in the unit that handles malaria test. Four doctors and one laboratory scientist were randomly selected, making a total of five interviewees for each centre.

### Focus group discussion participants’ recruitment

Participants were recruited from parents/caregivers who brought their children to the out-patient clinic. The screener question was asked individually to select parents/caregivers with desired demographic profile (understands Igbo language) and experience (a child or dependent treated of malaria in a tertiary hospital within the preceding six months). Potential participants were promised that their children will be seen immediately after the focus group discussion (FGD) to enhance their willingness to participate and sustain their cooperation till the end of the discussion.

### Pilot focus group discussion and in-depth interview

The essence was to be sure that the responses to the FGD and IDI guides yielded the relevant information. It also helped to refine the questions and identified grey areas that needed to be attended to. It also gave insight to the likely time that each discussion will last.

### Data collection methods

FGD and IDI guides were developed by a sociologist from the recommendations of the research team and the guides were reviewed. A four-item participant information form consisting of age, sex, occupation, and education was developed for the FGD. The participant information form was administered before commencing the focus group discussion and they filled their written responses. Both the participant information form and the discussion guide were written in English and then translated into Igbo. The Igbo language guide was reviewed by native Igbo speakers for clarity and easy understanding.

### Focus groups

Four focus groups were conducted at the two study sites; Federal Medical Center Umuahia and University of Nigeria Teaching Hospital Enugu, between April 23 and May 3, 2013. The groups were purposive sampled to be gender specific. All focus groups were conducted in Igbo language and lasted approximately between 45 and 60 minutes. The interviews were all tape recorded, notes taken and the tape records were later transcribed.

### In-depth interviews

In-depth interviews were with doctors and laboratory scientists working in the two tertiary hospitals. The IDIs were to find the opinions of medical professionals on issues concerning malaria diagnosis. In total, 10 in-depth interviews were conducted. Critical for IDI participant selection included that the respondent should be managing malaria cases. Purposive sampling was employed to select in-depth interview respondents. This was done in order to focus on people who have appropriate experience and insight in malaria care. The questions for doctors were divided into three topics with related sub-questions: (i) malaria diagnosis; (ii) malaria treatment, and (iii) follow-up. The questions for laboratory scientists were concentrated around malaria tests and challenges.

### Data analysis

The transcription was first written down under each sub-question and the commonly mentioned points were highlighted. These form the reported quotes.

### Ethical considerations

Ethical approval was obtained from the University of Nigeria Teaching Hospital, Enugu, Ethical Health and Research Committee. The aims of the study were explained to all the participants and recorded verbal informed consent were obtained before the interview started. The recorded information was handled with confidentiality, denying access to none research team members.

## Results and discussion

### Focus group discussion

Twenty-nine people participated in the four focus group discussions. Out of this number, 17 (58.6%) were female and 12 (41.4%) were male. The age range of the focus group participants was 25 to 46 years. The majority of the focus group participants were businessmen. The number of years of schooling completed ranged from 3 to 17. See also Table 1.

**Table 1:** Demography of the participants

**Variables**	**N = 29 (%)**
**Gender**	
Male	12 (41.4)
Female	17 (58.6)
**Age groups**	
25 to 29 years	6 (20.7)
30 to 34 years	10 (34.5)
35 to 39 years	6 (20.7)
40 to 44 years	4 (13.8)
45 to 49 years	3 (10.3)
**Occupation**	
Business	9 (31.0)
Civil servant	7 (24.2)
Unemployed/student	6 (20.7)
Farmer	4 (13.8)
Driver	3 (10.3)
**Education**	
6 years or less	2 (06.9)
7 to 9 years	2 (06.9)
10 to 12 years	11(37.9)
14 years and above	14 (48.3)

### Focus group discussion results

#### *Knowledge of malaria*

The participants were very responsive to the question, “What do you know about malaria and how a child with malaria will present?” The majority responded that, “Malaria is a major health problem in our country*.*” None mentioned *Plasmodium falciparum* as the causative agent. Several of them were able to mention common symptoms of malaria. The most common mentioned symptoms were: fever, weakness, poor appetite and vomiting. The following are prominent responses:

“A child with malaria will have his head hot, when you touch the body it will be very hot, especially the head area.”

“I know that when a child has malaria, he will be very weak, will like to lie down on the cold floor.”

“He will not eat; if you force him to eat, he will vomit it, wants to be drinking only water.”

“The child will be weak, with joint pain, cold, internal heat.”

#### *Other illnesses that can present like malaria*

There were quite few responses to the enquiry, “Are there other illnesses that can present like malaria?” The commonest amongst the responses was “*typhoid*” (enteric fever). Others included “*oyi*” (pneumonia), “*Iba ocha n’anya*” (hepatitis), and “*cholera*”.

#### *Health-seeking behaviour*

Self-medication was the most common response when asked, “What will you do if you noticed that your child has malaria?” They answered:

“*I will go to chemist (patent medicine dealer) and buy drugs.”*

“Any time my child has fever, I will go to pharmacy and ask them to mix drugs for me, multivitamins, paracetamol and blood tonic. If after two days my child is still ill, I will bring him to hospital.”

“Even if I will bring them to hospital, I will first give them something to cool down the fever before coming to the hospital. You know that children illness always start in the night.”

Only one respondent did not accept the practice of self-medication. *“Anytime I notice that my children are ill, I always take them to the hospital, children illnesses are always serious so I don’t like wasting time trying to treat them myself.*”

#### *Willingness to do malaria investigation if requested by a doctor*

Majority want laboratory investigation to be done for their children. When asked the question, “Which option will you choose when you see a doctor, to treat your child without investigation or do malaria test?” They answered:

“*I will like the doctor to do malaria test for my child to be sure of what is wrong with him.*”

“*Even if the doctor does not want to do malaria test, I will request that he do the test. Since I have treated my child at home and he did not respond. I will like the doctor to investigate to be sure that it is still malaria that is wrong with my child*.”

“If the investigation will be available the same day, I can wait, but to wait for 2–3 days without treatment, I will not do that.”

“At times I will do the test before seeing a doctor, so that he will know what is wrong with my child, and be able to write the correct drug.”

“It is entirely the duty of a doctor to take charge of management of my child’s illness. The request for laboratory test or not, lies with the doctor. I am not supposed to interfere.”

#### *Willingness to wait for malaria investigation result before receiving malaria treatment*

When the participants were asked “Will you be willing to wait for the malaria investigation result to be available before starting your child on anti-malarial drugs?” They were divided in their responses, although some said outright “no,”

“*How can I wait and watch my child dying, because I am waiting for malaria test result that will be ready in about 2 days’ time, I can’t do that, If the doctor refuses to start my child on treatment, I will look for a place where he will be treated.*”

“*It all depends, if my son’s illness is serious, the doctor must start him on something while waiting for the result, at least something to cool the fever*.”

Others were of the opinion that if the doctor explains and assured them that their children illnesses will not worsen while waiting for the malaria investigation result before commencing anti-malarial drug, they will wait.

“Since my child had received self- medication without improvement before coming to this hospital. I have to exercise patience if am asked to.”

“*If the doctor tells me to take my child home and come back another day for treatment, I will obey the doctor. Doctors are more knowledgeable about what is wrong with my child. But he must assure me that nothing will happen to my child if I take him home*.”

“*A drug must be given, but if the doctor insists I will obey him*.”

“*I will obey the doctor, this my daughter I brought to hospital today, I brought her on Tuesday (two days ago) and the doctor sent us to do test and to come back today without any treatment, no paracetamol, no antimalarial, nothing. I did as he instructed, today when I show him the result he (doctor) will decide on treatment.*”

“*I will plead with the doctor to start treatment for my child. If I go without treatment to come another day, I will likely be seen by another who doesn’t know what is wrong with my child and will start all over to ask me questions*.”

#### *Whether their prescriptions/diagnosis were explained to them*

The majority of the participants responded “no” when asked, “Do you know the content of doctors’ prescriptions for your child, or do doctors explain the prescription to you?” Some of their specific comments included:

“*Doctors do not tell people what is wrong with their children and they always write their prescription such that patients cannot read it.*”

“*Doctors never volunteer to explain their diagnosis.*”

“*I once asked, and the reply I got was, “If you know the diagnosis of what importance is it to you.*”

“*I brought my child the other day to the hospital, while I was in the hospital, my people (relatives) were calling me to know what was wrong with him, but I could not tell, since the doctor did not explain anything to me. Only that my son was receiving treatment and was getting better, that is all.*”

“*I am not bothered about what my child is on, once I can notice improvement I am ok. I know the reason why doctors write their prescription the way they do but I won’t say it.*”

“*They do not inform patients about their illness.*”

“*Doctors’ prescriptions are always very difficult to read.*”

#### *In-depth interviews results/Doctors*

The in-depth interviews enquired how you expect a child with malaria to present.

All the respondents accepted that malaria has different and no specific symptoms and thus can mimic many other illnesses. “*Fever, vomiting, chills and refusal to eat are the main symptoms and the common differential diagnoses of malaria are; pneumonia, urinary tract infection, acute gastro enteritis (AGE)*.” (Registrar, female, 5 years of practice).

“*Fever, joint pain, vomiting, headache (for children old enough to complain), and malaise are the common symptoms of malaria and acute respiratory tract infection and urinary tract infection were the closet differential diagnoses of malaria*” (Consultant Paediatrician, 15 years of practice).

#### *How do you decide on malaria?*

“*Malaria is endemic so should be considered first”* (Registrar, female, 5 years of practice).” “*Clinical diagnosis is important because laboratory results are not available the same day, thus the only reasonable option is presumptive treatment*” (Consultant Paediatrician, 15 years of practice).

“*Most parents that have treated their children but with no improvement, will request for investigation, but have never tried not starting medication due to lack of result*” (Consultant Paediatrician, 15 years of practice).

“*Mild cases can wait, but severe malaria cases always request for treatment first” “Endemicity of malaria makes it the first option to treat once suspected,”* (Registrar, male, 5 years of practice).

“*I always assume that it is malaria and you cannot be wrong. This is the tropics*.” “*Once I exclude other systems, I will treat for malaria.*” (Registrar, female, 6 years of practice).

#### *World Health Organization (WHO) Recommendation of confirmed malaria treatment*

“*Parental pressure to see improvement in their child’s illness, compel doctors to act quickly to commence treatment before getting results.*” “*Relying on laboratory result before treatment will give the parents the impression that the doctor is incompetent, thus there is a high possibility of the patient to seek care from an alternative source.*” (Registrar, female, 5 years of practice).

“*Rapid diagnostic test kits (RDTs) are not available in the clinics to facilitate confirmation of malaria before treatment*.” *“I may send for investigation, but I don’t delay my treatment for results to come.”* (Registrar, male, 5 years of practice).

“*Laboratory investigation has its drawback. It takes a minimum of 48 hours to have results and the patient before you needs attention. So why wait.*” *“Rapid diagnostic kit is not used. Though RDT is available, we do not use it. It was once tried and all the cases it was tried on were negative. Some of the negative RDT tests turned out positive when microscopy was done on them. This raised a lot of doubt on RDT. So for RDT to be used there is need for expertise.”* (Registrar, male, 5 years of practice).

“*I practice presumptive treatment most of the time. Rapid diagnostic test is not available in the clinic. Microscopy result will not be available till next day. Remember, these patients must have waited for a long time in the clinic before being seen by a doctor. Causing them further delay because of test result, does not go well with the patient.”* (Senior Registrar, female, 9 years of practice).

“*Siting of the hospital is not helping matters, most caregivers will not readily accept to leave without medication. They will always plead for medication. More so, there is the issue of cost of their transportation and inconveniences, if they are to come back another day*.” *“Rapid diagnostic test kit is available but only at the children emergency room, which is located at the other extreme of the hospital, there is none in this consulting area, thus I do presumptive treatment based on clinical assessment. I cannot rely on laboratory test.”* (Registrar, male, 7 years of practice).

“*It depends on my assessment of the patient; if acutely ill, I will treat immediately but if an uncomplicated case, it can wait for the test result. But since I cannot guarantee who will come back. I am always tempted to treat*.” “*Rapid diagnostic test kit is only available in the emergency room, but we don’t normally send them to emergency room to do the test*.” (Registrar, female, 6 years of practice).

#### *Explain prescription to patients*

“*I do but not always*” (Registrar, female, 5 years of practice).

“*I do inform my patients on how to take their medication.” “Sparingly due to busy clinic, but for mothers that request, I always oblige them.” “No I don’t.”* (Registrar, male, 6 years of practice).

#### *In-depth interviews results/Laboratory scientists*

According to most of the respondents, the laboratory is being over-worked. No single unit handles only malaria test. The shortest period malaria test can be ready is after 24 hours, thus malaria microscopy test cannot be done as urgent test. They also indicated that malaria tests are done in batches, so even when a test is sent early, it will not be started until a particular number of other malaria tests are collected. Additionally, they informed that rapid diagnostic test kits are not available in the laboratories.

“*The hematology unit that handles malaria test also takes care of other investigations like complete blood count, reticulocyte count, urgent hemoglobin estimation and erythrocyte sedimentation rate*.”

“*The capacity of the unit in terms of staff strength does not match with sample load.*” “*A minimum of about 70 samples out of which 50 may be for malaria test are handled every day. Our target is to get all results out within 24 hours. But due to logistics of records, patients may not be able to access their results until the second day*”

Responding to whether malaria test can be done as an urgent test, the respondents did not quite agree with that.

“*Malaria test cannot be urgent. No matter how fast a doctor wants it, the test has to pass through a standard process which takes at least, three hours.*”

“*We run malaria test in batches, so even when a request for malaria test comes early, it still has to wait until a sizeable number of other malaria tests are collected. One particular sample cannot be singled out for investigation.*”

“*If a physician brings the test request and the sample to the laboratory, it may attract specific attention, but writing “treat as urgent” is not considered. Imagine when virtually all investigations have “urgent” remark on them, it is hard to make out which one is really urgent.*” “*The habit of requesting for malaria test along with hemoglobin estimation or full blood count all in one form causes some delay. Because when the sample arrives, it is first sorted out based on the test options requested. So if only malaria test is requested for in a form, it eases the delay. More so, it can be sent directly to parasitology unit to handle and pressure of work over there is lighter, although this arrangement has not been fully worked out*.” “*No malaria rapid test is done here, although it has been recommended.*”

## Discussion

This study reported the perceptions of patients, doctors and laboratory personnel regarding the different factors that contribute to the presumptive treatment of malaria. All the parent/caregiver respondents agreed that malaria has many non-specific symptoms, which can also be manifested by other disease entities. This is similar to what was reported by other studies [19], where most of the respondents were knowledgeable of symptoms of malaria, as well as other illnesses that can present like malaria. It was documented that virtually all the respondents, except one had self-medication before presenting at a tertiary hospital. This practice, on the background of nonspecific symptoms of malaria means that parents and caregivers are at liberty to use different types of medication which may include anti-malarial drugs. This is supported by different African studies that have reported that about 90% of fever assumed to be malaria received treatment at home Uzochukwu *et al. *[16], Thera *et al. *[20], Fawole *et al. *[21], and Nyamongo *et al*. [22]. Their decision to visit the hospital or clinic is taken when the self-medication had failed to yield any appreciable effect. The reasons can be either; inadequate anti-malarial therapy or treating non-malaria illness with an anti-malarial. The latter is more likely to be the case, considering that since the introduction of ACT as the primary malaria treatment, the most anti-malarial drugs are sold in their packages with clear instructions and graphics on its dosage with regards to weight and age. Therefore, the adequacy of self-medication is to a greater extent guaranteed, compared to the era of chloroquine, sulphadoxine/pyrimethamine (SP) whereby the patent medicine vendors (PMV) purchased them in tins and later decided on the dose considered appropriate for the child, coupled with their poor knowledge of illness management [23]. In view of the above, it is most likely that adequate anti-malarial therapy should have been taken before presenting to the health facility. The possibility of persistent fever being malaria-related is less.

All the respondents were willing to do malaria test if prescribed by the doctor. There were some that affirmed that even if malaria test was not requested, they will ask for it. One obvious reason for this is that most of the respondents opt to use formal health care providers when their self-medication had failed. Thus, at this point they are very willing to know what is actually wrong with their children. However, virtually all the respondents would want their children to be started on medication while awaiting the investigation result. This may be because of the fact that an appreciable number of the respondents showed confidence in the providers and ready to comply with management plan, a disposition that is borne out of the perception that doctors in tertiary hospitals have higher level of formal training and experience [24]. Nonetheless, only one respondent accepted to comply if doctors decided not to start treating her child until investigation result is available. The providers’ perceptions of patients’ expectation of a prescription was the strongest predictor of their decision to prescribe as noted by Britten *et al. *[25]. Although patients want prescriptions, this need is based on their anticipation of what will improve the health condition of their sick child. Therefore, providers who still want to practice laboratory - confirmed treatment of malaria can institute symptomatic treatment with antipyretic, haematinics, and multivitamins, for uncomplicated cases on an out-patient basis until their malaria status is established [26]. For patients with features of complicated malaria, they can be admitted on supportive care with blood transfusion, correction of hypoglycaemia, anticonvulsants and fluid therapy, until malaria is confirmed and treated. It has been shown that the institution of early intensive care reduces mortality in patients with *P. falciparum* who did not show clinical response to anti-malarial treatment within 48 hr [27], it therefore means that with appropriate supportive care, commencement of anti-malarial treatment can be delayed for a while or rather till it is deemed fit. In this situation, the healthcare provider can make a special request by accompanying the blood sample to the laboratory for urgent and prompt attention.

There is no doubt whether children being managed on out-patient basis will return for further evaluation. Since in this study, it was shown that respondents are willing to return for the result of their tests and also for addition of more medication at a later date, even if their children have made remarkable improvement. Also, their knowledge that other illnesses can mimic malaria is an attribute that can be harnessed by health providers to encourage parents and caregivers to wait for proper evaluation before administrating anti-malarial therapy. The practice of not explaining prescription to parents that was complained about by most respondents in this study should be discouraged. Rather Doctors should make every effort to explain their management plan to patients, so that in the event that their children improved on supportive care, they would aspire to represent for final decision. Explaining their treatment plan will immensely enhance the parents’ knowledge about their health with regards to their choice of self-medication.

In this study, presumptive treatment is commonly practiced by all the providers. A finding similar to what Hamer *et al. *[28] reported in their study. This undermines the increasing body of evidence that greater percentage of febrile patients do not have malaria, especially now that studies from different localities indicate decline in malaria prevalence, Tagbo *et al*. [9] and Reyburn *et al*. [29]. Apart from the erroneous mindset that malaria is still hyper-endemic in Nigeria, there is also the misconception that relying on laboratory result is a salient indicator of incompetence on the part of the provider. What the providers with such mind-set should realize is that most respondents strongly support doing malaria test, especially when being managed in a tertiary hospital. But what they were reluctant to accept is delay in instituting treatment. Since treatment is not synonymous with the use of an anti-malarial, providers are at liberty to use their discretion on how to handle individual child. Considering that malaria test with microscopy takes time to be ready and RDTs are not made readily available in the consulting rooms, it is importance to device means through training and seminars to constantly remind providers of the prevalence of positive malaria parasitaemia among children with fever.

In this study, report from the laboratory section made it clear that the pressure of malaria test request exceeded the capacity of the unit that handled malaria test, mainly due to dependency on malaria microscopy, which cannot adequately satisfy the daily malaria test requests. If this remains the situation, it means that confirmation of malaria on the same day of request depending on microscopy is virtually impracticable in the tertiary hospital. On few occasions, if the provider brings the test request physically and makes a special request on it, will it be singled out and processed. A privilege all malaria tests cannot enjoy.

Thus, the only practical means to confirmed treatment of malaria on day zero is the deployment of RDTs to clinics and encouraging providers to use and rely on the results so obtained. There is need for proper storage of these kits and their being used before the expiration date to ensure their proper function, there is enormous evidence about the sensitivity and specificity of RDTs for diagnosing malaria [30,31]. Ironically, the lack of trust report by some of the provider respondents that have used RDTs, about the high negative results observed amongst children with fever. Could the low yield of positive result observed be evidence of the reported decline in positive malaria parasitaemia amongst febrile children from most districts where RBM is being implemented? [32].

### Limitation of the study

The study was done in only two tertiary hospitals in two different states. Although patients that visit the two hospitals come from diverse ethnicity and socioeconomic groups, the structure of the hospitals differ. Although there is very high chance that the problem existing in these two tertiary hospitals will be the same in other hospitals, there is need to identify the peculiar problems with the other hospitals in that region.

## Conclusion

This study highlights that as long as the confirmation of malaria in tertiary health facilities depends on microscopy, the practice of confirmed treatment of malaria on the day of presentation will remain an illusion. Nonetheless, providers can use their discretion to achieve some form of confirmed treatment of malaria, even if it means delayed inclusion of anti-malarial in the adjunctive therapy the child is already started on. Try as much as possible to explain their management plan to the parents, who have shown willingness to adhere to it. Deployment of RDT should be pursued, with quality control measures to guarantee confidence in its results.

## Abbreviations

ACT: Artemisinin-based combination therapy; FGD: Focus group discussion; IDI: In-depth interview; PTCM: Presumptive treatment of childhood malaria; RBM: Roll back malaria; RDT: Rapid diagnostic test kits; WHO: World health organization.

## Competing interests

The authors declare that they have no competing interests.

## Authors’ contributions

UMD conceived and designed the study. All the authors participated in data collection and analysis. All the authors made input in the writing of the manuscript. All authors read and approved the final manuscript.
